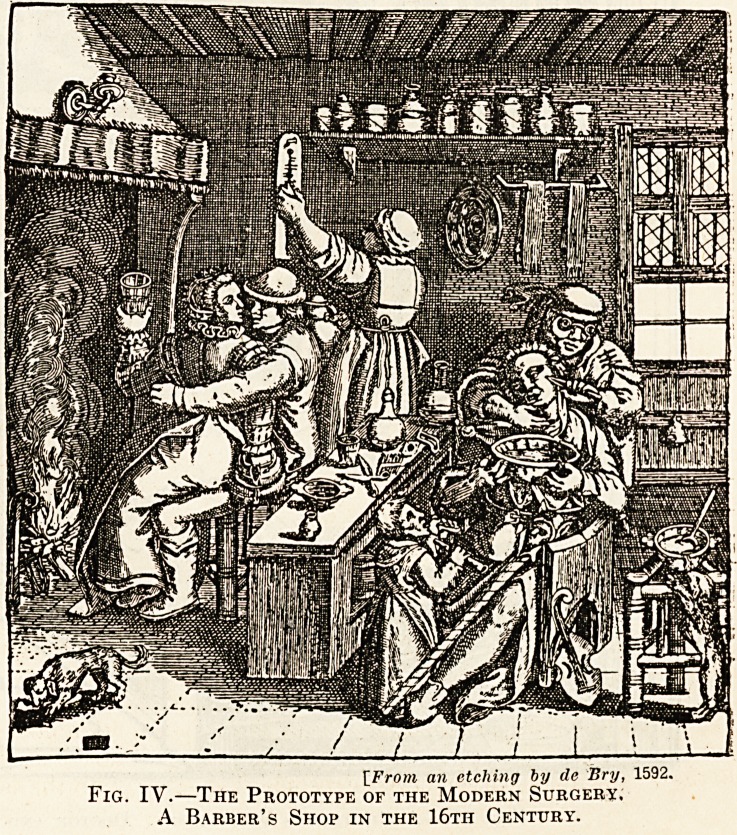# Christmas Appeal Number

**Published:** 1908-12-19

**Authors:** 


### CHRISTMAS APPEAL NUMBER.

### Hospital Christmas in the Past.

From the earliest times the yearly advent of Yule-tide has been eagerly anticipated by most hospitals in England and elsewhere, although it is only during the last half century that, with us at least, the word Christmas has developed into an institution. In the olden times, when hospitals were largely controlled by the clergy, feast and saints' days were strictly observed. Patients as well as physicians had their patrons in the calendar, as can be seen from the old engravings, and from the popular folk songs and rigmaroles of the middle ages. There was a patron saint for those afflicted with diseases of the eyes, with palsy, with rheumatism, with erysipelas, and, curiously enough, with laziness. The last saint still figures in the calendar, where the curious may find a special patron for those who cannot get up early in the morning. The major saints invoked by the profession were Saints Damian and Cosmo, physician martyrs in the second Roman persecution; St. Luke apostle, physician, artist, and presumably martyr, and St. Pantaleon, leech and the special protector of oculists. By the patients: St. Modwena, one of the few British saints in the calendar, a lady doctor, and a woman whose life's history is exceedingly

[*From an old German woodcut.*

Fig. I.—SS. Cosmo and Damian, Patron Saints of the Medical Profession.

[*From an old woodcut*, 1531.

Fig. II.—A Christmas Visitation. Doctor and Nurse at Bedside.

interesting to laymen and members of the profession alike; St. Lucy, St. Rochus (who was made the special patron of those afflicted with plague and

[*From a woodcut by Albrecht Durer.*

FIG. III.—CHRISTMAS FESTIVITIES IN A BATHING SANATORIUM.

afterwards with the "French disease" or syphilis), St. Phillip Benozzi, St. Vincent de Paula, St. Vitus, St. Ottillia, St. Giles, St. Adrian, St. Blaisé, and many others. Their days were observed with feasting or fast, according to the custom that rules such celebrations, in the various monastic establishments where the sick were cared for, in the "bathing sanatoria," the forerunners of the modern spas, and even in the barbers' shops, which represented the private surgery of the modern general medical practitioner.

Christmas had no particular interest to physicians, however much to patients. The former visited the latter once instead of twice during the day, as the old Salernian canon decreed, and gave medicines only in extreme cases. Merrymaking and feasting were allowed, in some cases with disastrous results, to judge from the complaint of the elder Copho, who remarks that "overeating at the feasts near the year's end is deadly to many afflicted with urinary trouble," from which one may presume that even in those days the profession had already damned the plum-pudding and the mince pie! Musical festivities were apparently "greatly beloved," and in an account of Master Alaric Sardanapalus, a physician to whom the profession is indebted for nothing of scientific interest, it is recorded that "dancing and the singing of lusty songs benefit patients with the wind and the throat spasm" (whatever that might be) "wherefore such are less in need of assistance at Christmas and Easter." The December feasts were kept at a very early period in Italian hospitals, where even to-day curious old customs are observed in the wards on Christmas-eve. In England it reached the establishments for the sick at a comparatively late period, though the New Year and Easter were observed even in the lazar houses from very early times. It would be an interesting study to investigate the history of the hospital Christmas in various countries, but at present no such investigation has been done, and one can only speculate on the probable events three hundred or more years ago from the stray allusions which are made in old chronicles.

The good bishop who established one of the first of Christian hospitals also incidentally interested the Nicean Council in the conditions under which the sick suffered, and got the reverend fathers to decree a special collection on certain Sundays in the year, such collection to be used for the benefit of the sick. This was the first Hospital Sunday fund of which there is any record, and its collection was carefully controlled until theological politics ousted the purely charitable spirit of the early Church fathers. Here and there, however, the Bishop's collection, as it was called, was still kept up. Special donations were set aside in lazar boxes which were opened at Christmas, and the contents distributed by the bishop himself to the lepers and cripples. As this custom led to some abuses lazar boxes were gradually abolished, and it was left solely to the

[*From an etching by de Bry,* 1592.

FIG. IV.—THE PROTOTYPE OF THE MODERN SURGERY. A BARBER'S SHOP IN THE 16TH CENTURY.

vicar or priests' discretion to give church alms to the sick out of the common church fund. The annual hospital sermon (which has been revived in many countries) was also discontinued, but in Austria and Poland a Christmas service was held for the sick, and in some places a special Christmas communion was held for their benefit.

As hospitals passed out of immediate ecclesiastical control the saints' days and feasts were honoured more in the breach than the observance, though in some countries the old customs remained. Thus in Russia even to-day there are many saints' days which are religiously observed in all the hospitals. The patients have a holiday. There are services conducted with all the solemnity of the Greek ritual, and the hospital is closed to visitors and even to the staff except for attendance upon extreme cases. In France, on the other hand, there are few days of festivity in the hospitals. The Revolution abolished the Christmas enjoyments and fixed nothing in their stead. Gradually, however, France is coming to regard the restitution of the Yule-tide pleasures as desirable, and in a few years' time the hospital Christmas in France will doubtless be as great an institution as it is at present in some of our hospitals.

### THE PUBLIC AND THE HOSPITALS.

As Christmas time comes round each year those concerned with the management of hospitals have a double preoccupation. They are confronted not merely with their public and private efforts to celebrate the great Christian festival of the year, and with their duty to the institution on behalf of which they utilise the opportunity to ask assistance of the charitable: beyond this loom for them the end of their financial year, and questions as to how the hospital has fared during the past twelve months, and how it is likely to fare in the next twelve. Hence, the Christmas appeal and such benefactions as result from it possess only too often a peculiar significance. Subscriptions and donations are never, of course, unwelcome at any time of year; but just about Christmas they have an especially stimulating effect to those hospital committees which are faced (and few, indeed, are not) with the nightmares of defective revenues, overdrafts to bankers, and prospective closure of beds. The hospitals, in fact, live from hand to mouth, and that is why there are so many special appeals issued to the public on their behalf. Faced as they nearly all are with excess of outgoings over receipts, it is sheer hard necessity which drives them to send round the hat with such persistence.

And after all, if at this season it is not permissible and laudable to ask men and women to remember and to put in practice one of the most fundamental and reiterated portions of the teachings of the Founder of Christianity, we know not when it is. At Christmas the postman, the dustman, and a score of others who are paid fair and sufficient wages for their work throughout the year, expect a "Christmas box," and get it. Have not the hospitals a greater and higher claim than this? Are not their labours of love, year in year out, placed freely at the disposal of suffering humanity, and may not they ask to be treated no less generously than those to whom the public owe no unpaid debts? We are not arguing that an occasional spasm of generosity at Christmas time is all that a well-to-do man or woman should regard as his or her duty to the voluntary hospitals. For those who can and do afford an annual subscription to a hospital (or to more than one) the question of an additional Christmas donation is one that may safely be left to their own careful consideration. But there is a much larger class of people who do not feel justified in setting apart annually beforehand certain sums for such purposes, yet are well able to afford substantial contributions unless any unforeseen financial difficulty should beset them. By these the Christmas appeal should be received with gratitude as a timely opportunity of well doing.

So also there is another very wide class to whom appeal may fittingly be made at this season; those, we mean, whose circumstances do not allow of annual subscriptions or donations of moment. To such people the existence of the voluntary hospitals means much; they are those who can afford to pay their private medical attendant for ordinary illnesses, but to whom a major surgical operation spells bankruptcy unless some help is forthcoming. They are often persons of great economic value to the community, young and industrious married folk, for instance, and to restore them to full wage-earning capacity is to render real service to the State. To this class the continued existence of the voluntary hospitals is a species of insurance against that misfortune of ill-health, which may temporarily overwhelm the most deserving. Small, individually, as may be the offerings which hospital authorities expect from this section of the alms-giving public, they would total altogether very considerable sums could the obligation be brought home to the beneficiaries. Some, it is true, are reached through the collections of the Hospital Sunday and Hospital Saturday Funds; but there are many thousands who are not reached at all. If this problem could be solved it would go far towards relieving many hospitals from the most pressing of their burdens. We suggest it, therefore, as a measure of practical philanthropy of no ordinary value, that those whose leisure permits—and few of us are so busy on Christmas and the next following days as to be leisureless—should extend their charity beyond their own Christmas gifts by offering to others the opportunity of contributing small amounts. The small tradesman, the superior artisan, the bank clerk, the teacher, and many more, are those to whom such appeals, personally introduced, may properly and reasonably be made on behalf of the local hospital, whether in town or country. Such collections should, of course, be attempted only with the express authorisation of the hospital committee, and should be conducted only with the assistance of some official document making clear that authorisation; otherwise a door is opened at once for fraud or the suspicion of fraud. If such a charitable appeal, which should be properly organised on a definite system if enough helpers are forthcoming in any one district, is for any reason distasteful, a similar object may be achieved by glee or carol parties, in which direction those of vocal or musical talent may offer their personal services in the good cause. It is indisputable that to render personal service in this way is more useful, as well as more in the true spirit of charity, than merely to give pecuniary assistance to a hospital; for it rouses many others, who have been indifferent or supine, to a sense of their duty, from whom, as the ever-widening rings round a stone dropped into a still pool, the moral effect of such awakening spreads in ever increasing ratio.

Let us put the matter in a slightly different way. The reason why hospitals are so much used, and so little supported by the classes that derive most benefit from them, is not want of heart, but want of thought. It was a very human characteristic attributed to the Prince of Darkness in the legend that "When the Devil was sick, the Devil a monk would be; When the Devil got well, the devil a monk was he," and the author of that rhyme was doubtless a close student of his fellow men. Yet it is not perversity that accounts for this attitude, but only forgetfulness. If then any can be found willing both to contribute according to their own means, and to help arouse from their lethargy others, it will be to the benefit of all concerned. And if any more entirely appropriate season of the year than that of Christmas can be suggested for such efforts, we should very much like to know when it is, and why it should be so considered.

### Hospitals with 150 Beds and Upwards.

### BIRMINGHAM GENERAL HOSPITAL.

The applicants during the past year have been so great that, in spite of the more chronic patients being sent to the Jaffray Branch Hospital and other convalescent institutions, there has been a constant list of patients waiting for admission. 5,568 in- and 62,102 out-patients have found relief during the past year. Last year there was a deficit in the annual income of £7,698. In a busy manufacturing town like Birmingham there is an obvious need for a large efficient general hospital. Besides the ordinary population, the factory hands are numerous, notably poor, and their very occupation renders them liable to sickness and accident. If the increased work of the hospital is to be continued, more income is absolutely necessary, and an earnest appeal is made for new subscriptions and donations. House Governor, Mr. Howard J. Collins.

### BROMPTON HOSPITAL FOR CONSUMPTION.

The work of this excellent charity is well known, and, during the past two or three years, great strides have been made in increasing its utility to the nation. Its premier position has been confirmed in a most striking manner by the award of the prize of 1,000 dollars offered by the International Congress on Tuberculosis recently held at Washington, in competition against all countries, for the best exhibit of the treatment of consumption in its more advanced stages; while the prize of 1,000 dollars offered for the best exhibit of the treatment of curable cases was divided between the Brompton Hospital Sanatorium at Frimley and the Whitehaven Sanatorium, Pennsylvania. Among the causes which have led to this gratifying success are the establishment of the Lady Almoner's Department, which keeps in touch with, and assists as far as possible, all needy and deserving cases. Many homeless patients and those suffering from unsuitable surroundings have been boarded out in the country whilst waiting for admission, and often given a fresh start in life after discharge. A scheme of voluntary notification which was set on foot by the hospital this year has also greatly added to its value to the public. As a result, the home of the patient, which in all probability has become a centre of infection, is brought under the direct notice of the medical officer of health. On the scientific side also the committee early recognised the value of the opsonic index in relation to the diagnosis of early cases of tuberculosis, and the hospital possesses fully equipped clinical laboratories for carrying out this and other scientific researches. The treatment by a system of carefully graduated exercises of the patients sent to the hospital Sanatorium at Frimley continues to meet with the most marked and encouraging results, a very large proportion of the patients being cured and discharged quite fit to return to their former occupations. Secretary, Mr. Frederick Wood, Brompton, S.W.

### HOSPITAL FOR SICK CHILDREN.

The present financial condition of this hospital leaves much to be desired, owing to exceptionally heavy expenses in connection with structural work which could not be longer delayed without incurring a still greater expense later on. As the result, it is in debt to its bankers to the extent of no less than £7,000. The amount of relief it now affords exceeds that of any previous year, but unfortunately the support extended to it has of late fallen off, and the utmost difficulty is consequently experienced in meeting current expenses. This hospital is the oldest and largest children's hospital in the British Empire, and patients are sent to it from all parts of the Kingdom and even the Colonies. The support, however, received from outside the Metropolis bears no proportion to the heavy expense thus involved, and it is felt that when this important fact becomes known immediate and substantial help will be forthcoming. At this particular season of the year it should not be difficult to arouse sympathy on behalf of a children's hospital, and it is therefore hoped that a liberal response will be made to this appeal. Secretary, Stewart Johnson, Great Ormond Street, W.C.

### KING'S COLLEGE HOSPITAL.

BUILDING in connection with the new King's College Hospital commenced early in March, and very rapid progress has been made. The block now being erected comprises the out-patient, casualty, bathing, electrical, and dispensary departments, and covers an area of an acre and a half. It is anticipated that the block will be completed within the contract time of 18 months, and before long it is hoped that the central or administration block will have been commenced. It will be remembered that the full scheme arranges for the erection of a hospital of 600 beds on the magnificent site of 12 acres on Denmark Hill, presented by the Hon. W. F. D. Smith, M.P., but the committee do not contemplate the construction of the whole hospital immediately. It is proposed to carry on the work uninterruptedly if possible until accommodation for 360 beds has been provided, and it is hoped to complete the full scheme at a later date as funds become available. It is of the greatest importance that the work should not be hampered through lack of funds. Contributions will be gratefully acknowledged by the Secretary, at the Hospital in Portugal Street, Lincoln's Inn, W.C.

### LONDON HOSPITAL.

THIS hospital is situated in the East End, where its influence for good is not confined to the medical relief it affords, for the humanising and civilising influences which result to a patient, from a residence within its walls, are of immense value to the citizens. Its work improves the character of large numbers of the population throughout one of the poorest districts of London. This may also be said with truth in regard to other hospitals, but the influence in the case of the London is more patent and appreciable owing to the vast numbers handled every year. The ordinary income, including donations, subscriptions, etc., is not much over £80,000, and the cost of upkeep is over £100,000. It would be a calamity to East London if the work of this noble institution had to be curtailed. Secretary, Mr. E. W. Morris.

### MIDDLESEX HOSPITAL.

THE general wards of this hospital accommodate 305 patients, and all treatment is entirely free. The assured income of the institution is totally disproportionate to the necessary expenditure, and, in consequence, it is mainly dependent on voluntary contributions for its maintenance. A unique feature of the Middlesex Hospital is its special Cancer Charity (forty-seven beds), where those stricken by this disease may obtain such treatment and relief from their sufferings as the highest medical skill, coupled with the sympathetic ministration of experienced nurses, can afford. The scientific investigation into the cause and cure of cancer has been systematically carried on since January 1900, and the fruits of these labours are periodically published in the "Archives" of the hospital. A branch hospital and convalescent home at Clacton-on-Sea relieves the general wards of convalescent patients, thus freeing the beds for patients who would otherwise have to be refused admission. During the past year 1,046 patients were benefited by the change to a bracing sea-air, and the continuance of careful nursing. These few facts serve to show that the Middlesex Hospital is eminently worthy of the liberal support of the public. Secretary-Superintendent, Mr. F. Clare-Melhado, Mortimer Street, W.

### MOUNT VERNON HOSPITAL FOR CONSUMPTION AND DISEASES OF THE CHEST.

FROM small beginnings this hospital has grown to a great national charity. Its two hospitals contain 220 beds, all of which are occupied. In addition the out-patient department in Fitzroy Square is largely attended, and is doing a useful and most important work. Some idea of this work may be gathered from the fact that patients are received from all parts of the Kingdom, and unhappily many have to be turned away because funds are insufficient to provide for them. It is engaged in a perpetual war against consumption, and is educating the public in regard to the prevention, as well as the cure, of a disease which is a veritable scourge. At the present moment a sum of £3,000 is required to carry on the work and to avoid the incubus of debt. Apart from the relief which the hospital affords, its work is of the greatest value to the nation, and deserves the support of every person who would wish to see consumption prevented and stamped out. Contributions may be sent to the Secretary, Mr. W. J. Morton, at the offices, 7 Fitzroy Square, W.

### NATIONAL HOSPITAL FOR THE PARALYSED AND EPILEPTIC.

A HOSPITAL that has a world-wide reputation, and is the largest of its kind, is the National Hospital for the Paralysed and Epileptic, Queen Square, Bloomsbury, which celebrates its jubilee in 1909. A well-known American physician, in sending a contribution to the jubilee fund, expresses his obligations to the National Hospital, and states that, in his opinion, the hospital and its medical staff have from the beginning constituted the neurological centre of English-speaking peoples, and have been far and away the most potent influence in neurological progress. He goes on to say, "What you have directly done for suffering humanity is beyond computation, but I believe that what you have done indirectly through the medical profession of the world is still greater." The jubilee scheme includes a much-needed extension of the out-patient department, in which the annual attendances of patients have increased over 100 per cent. in the last 25 years. A good feature of the scheme is that it will not appreciably increase the cost of maintenance; and this fact should commend itself to the attention of prudent almsgivers. Secretary, Mr. Godfrey H. Hamilton, Queen Square, W.C.

### ROYAL FREE HOSPITAL, GRAY'S INN ROAD.

This hospital was founded in 1828 on the principle of free and unrestricted admission of the Sick Poor; poverty and suffering being the only passports required. Having no endowment, it is entirely dependent on voluntary subscriptions, donations, and bequests. Over 2,500 poor sick persons are admitted to the wards annually, and advice and medicine is administered to about 40,000 out-patients and casualty cases, who resort to it, not only from the crowded courts and alleys in its neighbourhood, but from all parts of London and the suburban districts. The relief thus afforded costs about £16,000 per annum, while the reliable income does not exceed one-third of that sum. Moreover, it has been needful during the past few years to expend large sums upon various structural and other improvements, which have been necessary in order to bring the hospital up to the standard of modern sanitary requirements. With this heavy additional outlay the committee have been obliged to make strenuous efforts to avoid getting into debt, and they earnestly solicit increased support from the charitable public, so as to enable them to carry on the beneficent work of the hospital in an efficient manner. Subscriptions and donations will be thankfully received by the Treasurers, the Bankers (Lloyds Bank, Ltd., Holborn Circus, E.C.), or at the hospital by the Secretary, Mr. Conrad W. Thies, Gray's Inn Road, W.C.

### ROYAL HOSPITAL FOR INCURABLES, PUTNEY HEATH.

This is the most important institution of its kind, being the largest and longest established. Over 220 men and women suffering from incurable diseases are provided with a home for life within its walls, and pensions of £20 a year are granted to 700 who remain in their own homes. This splendid work costs nearly £35,000 a year, in spite of the close scrutiny that every item of expenditure is subjected to, and as invested funds bring in only £6,000, no less than £29,000 have to be secured. A Christmas appeal is being made in the hope that those who have sympathy for less fortunate creatures will liberally respond. An interesting booklet, "A Chorus of Celebrities," being messages from fifty well-known public men and women, with a number of new drawings by the foremost black and white artists of the day can be had on application to the Secretary, Mr. Charles Cutting, at the offices, 4 St. Paul's Churchyard, E.C.

### ST. BARTHOLOMEW'S HOSPITAL.

Since the opening of its new out-patient block a further step in the scheme of reconstruction of this hospital has been entered upon, and in the spring of the coming year will be opened a block of new buildings to be devoted to investigation in pathology and pharmacology, which are so essential in the daily work of a modern hospital. This hospital is doing a great work, but hitherto it has received no assistance from King Edward's Hospital Fund or from the Hospital Sunday and Saturday Funds; indeed it would appear to be suffering from its reputation as an institution not needing public aid. At one time the income arising from endowments was adequate, with economy, to defray the annual cost of its maintenance, but owing to various causes the expenditure now exceeds the receipts by some £10,000 per annum, and the Governors are considering what steps should be taken to meet this difference. Clerk, Mr. Thomas Hayes, West Smithfield, E.C.

### ST. GEORGE'S HOSPITAL.

This hospital is most urgently in need of additional subscriptions. It is thought that many people who might subscribe to this charity, refrain from doing so from the impression that, as it is situated in a wealthy neighbourhood, it is necessarily a rich hospital. The ordinary expenditure exceeds the ordinary income by £15,000 a year. To meet this the hospital is compelled to sell out stock. No wards are closed, but the whole of the beds are used to the best advantage for the public. Some years back the annual subscriptions amounted to over £8,200, whereas in 1907 they had fallen to £5,700. The number of in-patients treated in 1907 was 4,469, and out-patients 43,989. St. George's has the largest convalescent hospital attached to any individual hospital in England, containing 100 beds, to which over 27 per cent. of the in-patients in 1907 were sent, entirely without any charge. The hospital has a most complete electrical department, fitted with the Finsen light and $x$-ray apparatus, and everything possible that scientific research can accomplish is done for the alleviation of the sick. Secretary, Mr. H. Wingrove, Hyde Park Corner, S.W.

### ST. MARY'S HOSPITAL, PADDINGTON.

Few institutions can possess a more powerful claim to public support than that of the general hospital of Paddington. Whether that claim be based on the past record of the hospital, its present work, or its promise for the future, it is impossible to deny its force. Of St. Mary's past the *Times* in a recent article said: "It has had no time to build up venerable traditions, but from the day of its opening (1851) it has continued to hold a high place among its contemporaries and its reputation has been continually enhanced." With regard to its work in the present, authoritative statistics show that the work done by one bed on the average in this West-End hospital is greater and the expenditure in maintaining a bed less than in almost any other similar hospital in London. Concerning the future, may not St. Mary's be said to be the birthplace and nursery of treatment by therapeutic inoculation, the great possibilities of which are now receiving universal recognition? But, in spite of such claims as these, the hospital is inadequately supported. Last year ended with a deficit of £2,000, and there is reason to fear that this year will close with a debt of double that amount. If this fear be realised, the closing of beds cannot be far off, for there is no funded property to speak of. The public should see to it that this calamity does not befall the poor of Paddington and the surrounding district. Secretary, Mr. Thomas Ryan.

### SEAMEN'S HOSPITAL SOCIETY.

Some idea of the scope of the work of the Seamen's Hospital Society (*Dreadnought*), may be gathered from the fact that 30,000 sailors seek relief annually in the institutions of the charity from all parts of the world. When British sailors are disabled or taken ill in foreign ports, they are at once sent home by the Consuls to the *Dreadnought* at Greenwich, where they are accorded a kindly welcome and every medical comfort. In short, all ways lead to Greenwich, where the sick or disabled sailor is concerned. It is the oldest Marine Charity, and for nearly a century has rendered a great service to the Empire. It is entirely dependent upon voluntary contributions. £20,000 is needed annually to support the two hospitals and two dispensaries of the Society, and the committee most earnestly appeal to the public to spare at least something for sailors, to whose labour and bravery everyone is personally indebted. Contributions may be sent direct to the Secretary, Mr. P. Michelli, C.M.G., at the *Dreadnought* Hospital, Greenwich.

### UNIVERSITY COLLEGE HOSPITAL.

The present need of this hospital is very great. Repeated sales of investments to make good deficits have so reduced the funded property that it must soon be exhausted, and, as an inevitable consequence, beds will have to be closed. It will be a great misfortune if the poor should have to be denied the full benefit of Sir J. Blundell Maple's noble gift of the new hospital. Its annual upkeep costs about £28,000, and the annual charge of nearly 3,600 poor sick people in the wards and of over 50,000 as out-patients is a serious responsibility. The remedies and surgical appliances are costly; the experienced and well-trained nursing staff entail considerable annual outlay; and the dietary is necessarily on a liberal scale. It can confidently be stated, however, that the strictest economy has been maintained. The reliable income is £9,000, and consequently £19,000 has to be obtained in voluntary contributions every year. Deeply grateful for past help, the committee trust that their appeal may be liberally responded to, as this will give them encouragement in carrying on their work of charity. They also venture to urge the claims of the charity upon testators. Treasurer and Chairman, Mr. Henry Lucas, Gower Street, W.C.

### Hospitals with Under 150 Beds.

### CANCER HOSPITAL (FREE).

This hospital is the only special hospital in London for the treatment of cancer. The nursing of cancer involves great expense, for not only have the dressings and drugs to be supplied in liberal quantities, but the patients often need a very generous and expensive dietary. It will thus be understood what a terrible infliction it must be to the poor when cancer is developed in any member of their family, and what a boon such a special hospital as the Cancer Hospital is to them, where their dear one will receive not only the best medical and surgical treatment, but will also be nursed and fed in a manner far above their means to procure. The maintenance of such an institution is therefore necessarily most expensive, and the committee of management earnestly appeal for support, by annual subscriptions, donations, or legacies, in their endeavours to carry on the good work. The hospital is quite free, neither letters of recommendation nor payment being necessary, the only passports required being that the applicants shall be in necessitous circumstances and suffering from cancer, tumours, or allied diseases. Secretary, Mr. Fred. W. Howell, Fulham Road, London, S.W.

### GREAT NORTHERN CENTRAL HOSPITAL.

This is one of the most modern of London's hospitals, the present building having been constructed in 1894. It has 167 beds, and over 2,300 in-patients and 27,000 out-patients are relieved annually. The hospital has risen in a comparatively few years from a small beginning, and is now the largest of the general hospitals without medical schools. Of the £16,000 required annually to maintain it, only £6,000 can be relied upon. The hospital has this year experienced a very serious and quite unprecedented falling off of income, the receipts being more than £7,000 less than the outgoings. At present over £12,000 is owing to the bankers for advances on current account, and with Christmas bills to meet the financial outlook is serious. The funds of this hospital are carefully and economically administered; it is doing excellent work in a very poor district and deserves a larger measure of support. Secretary, Mr. L. H. Glenton-Kerr, Holloway Road, N.

### LONDON HOMŒOPATHIC HOSPITAL.

This is the only hospital in London conducted on homœopathic principles, and appeals to all interested in the care of the sick and suffering poor. It may fairly be classed as one of London's large general hospitals, with separate departments for special diseases. Last year it treated 1,105 in-patients and the out-patients numbered 10,167. For some time past the accommodation has been inadequate to meet the increasing demands, and last year the magnificent sum of £30,000 was raised for enlarging the hospital to the extent of providing 170 beds as against the present 104. This extension is now well in hand and when finished the institution will be one of the most complete and up-to-date hospitals in the Kingdom. The board are now earnestly appealing for £2,500 to furnish this extension. For nearly the whole of the annual expenditure of £9,000 the committee have to look to the generous public, and help by way of subscriptions or donations will be thankfully received by Mr. Edward A. Attwood, the Secretary, at the hospital, Great Ormond Street, W.C.

### METROPOLITAN HOSPITAL.

This institution is situated in one of the poorest and most crowded districts of London, and is therefore most suitably placed for its purpose, but in consequence of its being far removed from the neighbourhood of the well to do, it unfortunately does not receive the support it so much needs and so well deserves. Of its 113 beds 106 were daily occupied during last year, and it relieved 1,933 in-patients and 42,264 out-patients. During the last three years the average ordinary annual income has been £13,120, and the average ordinary annual expenditure £13,936. A special appeal is made for funds to put the Hospital into thorough repair, of which it stands in great need. Secretary, Mr. J. C. Buchanan, at Kingsland Road, N.E.

### POPLAR HOSPITAL FOR ACCIDENTS.

Situated among a teeming population of poor hard-working people in a district which may be called the "workshop" as well as the "port" of London, this hospital is doing an excellent work. The demands on the institution have of late years greatly increased, and consequently further support is very necessary, especially as the Committee, after long and anxious consideration, decided last year to build a convalescent home at Walton-on-the-Naze. This home was finished and ready for patients in June last, and the twelve free beds have been fully occupied since that date. The total cost of the land, building, and equipment has been £7,000, and the upkeep will cost about £800 per annum. Secretary, Mr. Percy Rogers.

### PRINCE OF WALES'S GENERAL HOSPITAL, TOTTENHAM.

The situation of this hospital is peculiar to itself. Tottenham has been described as the home of the unskilled, and one of the dormitories of London, where the dwellings of the poor appear to be hidden behind respectable houses, and, by thus escaping general observation, give rise to the impression that the district is capable of self-maintenance. As a matter of fact, there is little money there, and none for charity; moreover, prosperous business men have no occasion to go there as in the case of a manufacturing or shipping district. The result is that the hospital, which is dependent upon the help of the wealthy and benevolent, is very inadequately supported, and at the present is in debt to the extent of £7,000. Director, Mr. Frederick W. Drewett.

### QUEEN ALEXANDRA'S HOSPITAL FOR CHILDREN.

(Late North-Eastern).

This hospital is feeling the effects of the present widespread trade depression in a double sense. On the one hand, the increased poverty of the neighbouring districts causes such an accession of patients that the staff can barely keep pace with the work; while, on the other, the contributions have fallen off to such an extent that a heavy debt has been incurred, and the hospital will close the year with a very large deficit unless help is forthcoming. Great care is taken to prevent abuse of this charity, and the result of the committee's constant efforts in the direction of economy is shown by the fact that the weekly cost per in-patient last year was only 25s. 6d. The hospital has scarcely any endowment, and depends on voluntary contributions to the extent of £10,000 a year. It is extremely important that prompt assistance should be forthcoming to meet the present pressing needs. Secretary, Mr. T. Glenton-Kerr, at the Hospital, Hackney Road, E.

### VICTORIA HOSPITAL FOR CHILDREN, CHELSEA, S.W.

The record of 40 years shows how necessary this hospital is for the welfare of the poor children of South-West London. Yet it is a fact that comparatively few of the well-to-do residents of this district support it. Last year there were over 60,000 attendances in the out-patients' department; 1,008 admissions to the wards, and 550 to the convalescent home. It costs £8,000 a year to maintain the hospital and £1,500 for the convalescent home at Broadstairs, but as only £515 is derived from invested funds £9,000 has to be obtained from voluntary sources. At present strenuous efforts are being made to raise additional funds so as to prevent a large deficit at the end of the year. Secretary, Mr. H. G. Evered, Tite Street, Chelsea, S.W.

### Hospitals with Under 100 Beds.

### BOURNEMOUTH ROYAL NATIONAL SANATORIUM FOR CONSUMPTION AND DISEASES OF THE CHEST.

During 1907 patients were received from 33 different counties in England, besides others from Wales, Scotland, and Ireland. The marked success which has attended the hygienic or "open-air" treatment of consumption, together with the favourable situation and climate in which the sanatorium stands, lead many hundreds of poor sufferers to seek admission each year, and applications have increased so largely that it has been necessary recently to further enlarge the institution. There has been a great falling-off in voluntary contributions this year for ordinary purposes, and £800 is urgently required before the end of the year to meet current expenses. The ordinary annual expenditure has been permanently increased in order to maintain the additional patients for whom extra accommodation has been provided. For this purpose new annual subscriptions are necessary, without which it will be impossible to keep the whole of the sanatorium open. The committee, therefore, most earnestly plead for liberal assistance. Treasurer, the Hon. W. F. D. Smith, M.P.; Secretary, Mr. A. G. A. Major.

### CHELSEA HOSPITAL FOR WOMEN.

THIS hospital is completing a year unsurpassed in excellence of medical work, but, unfortunately, considerably below the average in financial prosperity. Its regular supporters, it is true, have responded with unfailing generosity to its claims, and where death or loss of income has diminished their number new friends have been forthcoming to fill the gap. But in legacies and substantial donations there has been a marked falling off. It is not sufficiently well known that £2,000 more than the reliable income is needed annually to meet the normal expenditure. The Council have always made a point of avoiding anything but necessary expenditure and of keeping clear of debt, but at present they are under the unusual and unpleasant necessity of incurring an overdraft of nearly £1,000 at the bank. It should be borne in mind that the nature of the work done at this hospital and the local circumstances make the cost comparatively high, but ample justification for the outlay is clearly shown by the statistics. Considering the severe and critical cases that frequently occur a rate of mortality of under 2 per cent. is remarkable. The Council trust that such work, admirably supplemented as it is by that of the Convalescent Home at St. Leonards-on-Sea, may not be overlooked by those able to place its finances in a more favourable condition. Contributions may be sent to the Treasurer, Mr. Henry E. Wright, or the Secretary, Mr. Herbert H. Jennings, at the Hospital, Fulham Road, S.W.

### HAMPSTEAD GENERAL HOSPITAL (with which is amalgamated the NORTH-WEST LONDON HOSPITAL).

THE amalgamated institution is now working on the lines agreed upon, the beds at the North-West London Hospital having been closed and the out-patients' department being continued there. This arrangement represents undoubtedly a very great advantage to the poor of Kentish Town, as they now enjoy the benefits of the healthful surroundings of the Hampstead Hospital. Considerable difficulty was experienced by both the institutions in obtaining an adequate income, but the claim to general support of the combined hospital will doubtless receive recognition. The hospital retains contributory beds in which local medical men attend their own patients. The needs of the combined institution are, however, very great, and there is an accumulated deficit of £6,000, besides which the necessary expenditure is no less than £5,000 in excess of the more or less reliable income. Secretary, Mr. George Watts, Haverstock Hill, N.W.

### HOSPITAL FOR WOMEN, SOHO SQUARE, W.

THIS hospital is the pioneer of women's hospitals, and the chief point to be remembered in connection with it is that while its work continues to increase, its income has steadily declined. There has been a deficit on the year's working for the last eight years, and the committee have had to borrow from time to time to meet current expenses. The mortgage loan now amounts to £7,000, which costs the hospital £290 a year for interest. The number of in-patients treated in 1907 was 924—the highest in the records of the institution. There were 3,943 new out-patients treated, whose attendances totalled to 16,334. It is lamentable that such a hospital should be so inadequately supported. It is hoped that relief will be speedily forthcoming to enable the committee to keep the wards open and pay off the mortgage debt. Secretary, Mr. Alfred Hayward, Soho Square, W.

### QUEEN CHARLOTTE'S LYING-IN HOSPITAL.

THIS hospital now receives over 1,700 poor women into its wards yearly for their confinements, and in addition attends over 2,000 women in their own homes. Not only are the in-patients fed, nursed, and provided with the most skilled medical attendance, but all the clothing for the mothers and their infants is also provided by the hospital. This entails a large expenditure, and the washing of the linen is still heavier. It costs over £6,500 per annum to maintain the hospital, but the reliable income is about £4,000 only. A great work is also done in the training of midwives, nearly all of whom enter for the certificate of the Central Midwives Board with very gratifying results. The receipts from donations and legacies have fallen off so much during the past two years that there is now a deficit of £2,000 on the general account. The sum of £7,500 is also needed for the Nurses' Home Extension which was recently opened. Secretary, Mr. A. Watts, Marylebone Road, N.W.

### SAMARITAN FREE HOSPITAL FOR WOMEN.

THIS hospital is quite free. It receives only poor women who are suffering from diseases peculiar to their sex; it is virtually unendowed, and dependent, therefore, upon the support of the benevolent for its existence. It requires £6,000 yearly for its upkeep. Although a Metropolitan hospital, it takes patients from all parts of the United Kingdom, and even from abroad; over 30 per cent. of them come from the provinces. In addition to the £6,000 required for maintenance, £3,000 is wanted at once to pay for a new operating theatre and other necessary improvements now completed. The committee earnestly plead for contributions to meet the heavy expenditure incurred in re-flooring one half of the wards and in renewing much ward furniture. New annual subscriptions are also earnestly solicited. Secretary, Mr. W. G. King, Marylebone Road, N.W.

### Hospitals with Less than 50 Beds.

### BRITISH LYING-IN HOSPITAL.

THIS hospital has carried on its excellent work for 155 years, taking patients from all parts of London and its suburbs. Like many other charities of this class, it has of late years received fewer legacies and donations than in the past, although its work and liabilities have increased. Much money has been spent on improvements to keep pace with modern hygienic and scientific demands, and the institution is now sadly in need of funds. More accommodation in certain directions is required in order that the pupils in the training school may obtain more conveniently the thorough education in maternity work, which is necessary for the safety of mothers and for the public weal. The committee will most gratefully acknowledge the receipt of subscriptions and donations, however small. Mr. A. C. Wickins, Secretary, Endell Street, W.C.

### CENTRAL LONDON OPHTHALMIC HOSPITAL.

SIXTY-FIVE years' good and useful work is the record of this hospital. It is situated in a poor and densely populated neighbourhood, and receives patients from all parts of the country. Last year there were 409 in-patients and 29,345 attendances of out-patients. The total expenses for maintenance amounted to £1,953, which shows that the important work of the hospital is being carried on at the least possible expense. There is no endowment, and new annual subscriptions and donations are much needed. The present buildings are old, and unsuitable for use as a hospital, and, as the lease will expire shortly, rebuilding has become absolutely necessary. The work is about to be commenced; £10,000 has so far been collected, but a further £10,000 is required to complete the work. The committee therefore make an earnest appeal to the charitable public. Secretary, Mr. H. R. S. Druce, Gray's Inn Road, W.C.

### CITY OF LONDON LYING-IN HOSPITAL.

THE committee make an urgent appeal for funds to liquidate the heavy debt incurred by the compulsory rebuilding of the hospital, the old one having been condemned as unsafe by the London County Council in consequence of the damage to the foundations caused by the construction of the "Tube' Railway close by. The rebuilding and furnishing have entailed an expenditure of £40,000, and, in order to partially meet this, the committee have been obliged to obtain a loan of £22,000 from the bankers, which, especially in the present state of the money market, is nothing short of a disaster to the hospital. The hospital is situate in one of the very poorest parts of the Metropolis, its patients being mainly drawn from the vast working-class population of Bethnal Green, Hoxton, Shoreditch, St. Luke's, and Islington. It has carried on this great work of mercy for the past 157 years. Some 3,500 poor women are delivered annually. A donation of £1,000 will name a ward; a donation of £100 will name a bed; life governor's qualification, £10 10s. It must be a source of gratification to those who are able to pay for their children being born in surroundings of happiness and comfort to know that by giving a donation to this hospital they are able to extend similar blessings to their less fortunate sisters. Secretary, Mr. R. A. Owthwaite, City Road, E.C.

### HOSPITAL AND HOME FOR INCURABLE CHILDREN.

THE accommodation at this hospital has been extended from 37 to 45 cots, and further additions will be carried out as soon as practicable. There are now more afflicted children than ever receiving the benefits of home and hospital which this Charity provides, but unfortunately this increased work is not accompanied by the necessary increase in support, and at the present time there is a pressing need for new subscriptions. An appeal on behalf of poor children suffering from incurable afflictions should meet with a most liberal response at this particular season. Honorary Secretary, Mr. S. Liddon Walters, North Court, College Villas Road, Hampstead, N.W.

### HOSPITAL FOR EPILEPSY AND PARALYSIS.

PATIENTS are received into this hospital from all parts, suffering from all kinds of nervous diseases, the great majority of the sufferers being inadmissible to general hospitals. It affords free treatment to the necessitous poor, but encourages all who are able to pay what they can afford, and thus promotes providence and minimises abuses. Its beds are constantly occupied and the attendances of outpatients average 13,000 per annum. The maintenance of the hospital for the current year will cost about £3,500, and as £3,000 has been received in income the sum of £500 is still required to avoid a deficit. Secretary, Mr. H. W. Burleigh, 4 Maida Vale, W.

### ST. JOHN'S HOSPITAL FOR DISEASES OF THE SKIN.

THIS is the only hospital in London or the neighbourhood especially built to meet the requirements of patients suffering from skin diseases. A new hospital is now open, and has a department for *x*-ray and other electrical treatment. Research work has recently resulted in a discovery of great value in the treatment of skin disease. The diseases treated at this hospital, which cause great suffering and involve prolonged and expensive treatment, do not appear to attract that sympathy which is accorded to other kinds of disease and distress, and, in consequence, this hospital comes very badly off in the matter of legacies and donations. There are ever-pressing demands on the charity for relief, and in order that this may not be misplaced, close inquiries are made as to applicants' circumstances. The sum of £7,000 is needed for the new building in Leicester Square, and an additional £8,000 to purchase the freehold of the in-patient department in Uxbridge Road. Secretary, Mr. G. A. Arnaudin, Leicester Square, W.C.

### General Charities.

### AFTER-CARE ASSOCIATION.

This is the only charity of the kind in the kingdom devoted to the re-establishment in life of poor persons discharged recovered from asylums for the insane. It appeals for funds to carry on and extend the work, the objects of which are to assist in various ways recovered cases on their discharge from asylums. Secretary, H. Thornhill Roxby, Church House, Dean's Yard, Westminster, S.W.

### BRITISH ORPHAN ASYLUM, SLOUGH.

This Charity was instituted in the year 1827 for the maintenance and education of fatherless children of once prosperous parents. There are always more than two hundred boys and girls in the schools, who receive a superior education, regulated by the requirements of the Cambridge University Syndicate for Local Examinations, and sound religious training, whereby they are qualified to attain the social position once occupied by their parents. The annual income from subscriptions and invested capital falls short of the necessary expenditure by some £4,000. There is no endowment, and a very earnest appeal is made for the increased support which is required to carry on unimpaired the good work of this old national charity. Secretary, Mr. C. T. Hoskins, 27 Clement's Lane, London, E.C.

### FREE CONVALESCENT HOMES.

Anyone visiting the wards of a hospital, and seeing the weary-stricken faces lying there, would realise the necessity of a thorough change of air and scene to completely restore health. Since 1840 the Metropolitan Institution has, by providing free convalescent homes, been administering to this necessity. It now comprises four homes containing a total of 359 beds, and receiving annually over 7,500 patients. About £14,000 is needed yearly to maintain these homes, to which patients are admitted entirely free of charge upon the recommendation of subscribers, and nearly the entire amount has to be obtained from voluntary sources. In addition, a sum of £7,000 is required for the completion of the new home, for men only, at Little Common, which was opened in 1905. To free the institution from these burdens an increased number of donors and subscribers is needed. Secretary, Mr. Alex. Hayes, 32 Sackville Street, W.

### LONDON FEMALE GUARDIAN SOCIETY.

This Society was founded in 1807, for the rescue of fallen women from all parts of the United Kingdom, with free and immediate admission to all applicants, and is therefore national in its work and influence. Many thousands of betrayed and helpless women have been rescued from the streets, welcomed to its homes, shielded from evil, instructed in the way of righteousness, and freely supplied with food, clothing, and a long residence of 18 months, for training as domestic servants, while the Society has advised, helped, and sheltered some thousands of casual and necessitous cases. The Homes can accommodate about 90 cases and are dependent entirely upon the charity of the benevolent, having no endowment. Secretary: Mr. W. E. Page, 191 High Street, Stoke Newington, N.

### LONDON ORPHAN ASYLUM, WATFORD.

No less than 6,886 orphans of the necessitous "middle class" have received maintenance and education from this charity, being afterwards placed out in situations. There are nearly 500 children at present in the school from all parts of the British Empire, and a great many seeking admission, whom the managers would admit in January if the necessary public support could be relied upon. Of the annual expenditure, £14,000 is required from voluntary sources. We trust that the present season will bring new friends and generous support, as the institution is benefiting a class peculiarly susceptible to the privations of adversity—privations too often borne in silence and untouched by the wave of popular charity. The example and teaching of the supreme Lover of humanity has left no room for doubt that to the best of our ability it is a duty to succour, as well as pray for, "all those who are desolate and oppressed." Secretary, Mr. H. C. Armiger, 3 Crosby Square, Bishopsgate, E.C.

### NATIONAL CHILDREN'S HOME AND ORPHANAGE.

This is one of the largest of the child-saving institutions in the country, and may claim to be a national work. It is fettered by no hard-and-fast rules, and a child's admission is decided irrespective of creed. It is really an association of homes under one management. Besides the head quarters in London there are important branches in Lancashire (Edgworth, near Bolton), Birmingham (Princess Alice Orphange), Farnborough, Hants (Certified Industrial School), Cheshire (Frodsham), Alverstoke (Hants), and Isle of Man (Ramsey). There is a convalescent home and cripples' hospital at Chipping Norton; a small village home at Chadlington (Oxon), and an emigration home in Hamilton (Ontario). New branches are about to be established at Harpenden (Herts), and at Bramhope, near Leeds. A sanatorium for children threatened with consumption is also being built on the estate at Harpenden. In all the branches the children live in small families under the care of "sisters," several of whom give their services without remuneration. The children receive a good elementary education and industrial training. More than 2,000 children have been emigrated, and double that number have been placed in situations at home. More than 8,000 have been benefited by the Institution, and about 2,000 are now in residence. Full information can be obtained from the Principal, Rev. Dr. Gregory, Bonnor Road, N.E.

### PLAISTOW CHRISTMAS DISTRIBUTION.

On Thursday, December 24, at 12 noon, in St. Mary's Parish Hall, Plaistow, E., there will be a distribution of joints of beef, materials for plum-puddings, bread, potatoes, coal, and warm clothing to poor families, who have been selected without distinction of creed for these Christmas gifts. The Rev. T. Given-Wilson earnestly appeals for funds in order that at least 1,000 of the poorest families in his parish of 20,000 may have their desolate homes made bright and warm with Christmas cheer on that great festival of peace and goodwill. Cheques may be paid to Union of London and Smiths Bank, 50 Cornhill, E.C., or direct to Rev. T. Given-Wilson, vicar of Plaistow, London, E. Visitors will be welcome.

### ROYAL ASSOCIATION IN AID OF THE DEAF AND DUMB.

The committee of this Association solicit contributions to enable them to give the usual Christmas gifts to the deaf and dumb poor. Last year on Christmas Eve 381 of these poor afflicted people were supplied either with provisions or gifts of money, and on Christmas Day 36 men and youths were supplied with a substantial dinner. A large number were entertained to a hearty tea and an evening's amusement at meetings held in the different districts. Any balance that remains after the Christmas gifts have been provided for will be transferred to the general fund. To encourage provident habits among the deaf and dumb a penny bank has been established, and help is solicited to enable the committee to give the promised interest. Besides these special objects, the committee are constantly giving pecuniary assistance to the deaf and dumb in sickness and distress. During the past year 3,240 visits have been paid to the deaf and dumb. Employment has been found for 185. There are 22,000 deaf and dumb persons in the United Kingdom, about 3,000 of whom reside in London, forming an isolated community incapable to a great extent of entering into the ordinary social relationships of life, or to plead on its own behalf. Sympathy and help from those who enjoy the invaluable gifts of hearing and speech are requested for the Association and its work. Secretary, Mr. Thomas Cole, 419 Oxford Street, London, W.

### ROYAL MATERNITY CHARITY OF LONDON.

This charity provides midwives and medical attendance for poor married women at their own homes free of charge by means of subscribers' "letters." Upwards of 3,000 patients are annually attended at their confinement, and for ten or more days afterwards. The personnel and equipment of the charity has been thoroughly reorganised and brought into line with the latest modern requirements. It is an anxious period, even in homes with every luxury, and in those where abject poverty prevails it is beset with danger, and calls for skilful aid and sympathy. The amount of relief given has had to be reduced through loss of income, and £2,500 is owing to the bankers, which seriously hampers the work. Major G. L. B. Killick, Secretary, 31 Finsbury Square, E.C.

### THE MARY WARDELL CONVALESCENT HOME FOR SCARLET FEVER, STANMORE.

The valuable work which this home is performing is not realised, or it would receive a larger measure of support. Were it not for this institution there would be no place to which to send convalescents from scarlet fever. It has benefited over 4,800 patients. Though originally intended for the working classes, it also admits patients from the upper classes. The fees paid are not nearly sufficient to maintain the home. A debt of £400 has been incurred in carrying out repairs which have been postponed for ten years through lack of funds, and there still remains to be paid off a loan of £200. The committee now plead for help, and especially solicit annual subscriptions. Miss Mary Wardell, Hon. Secretary, Stanmore, Middlesex.

## Figures and Tables

**Fig. I. f1:**
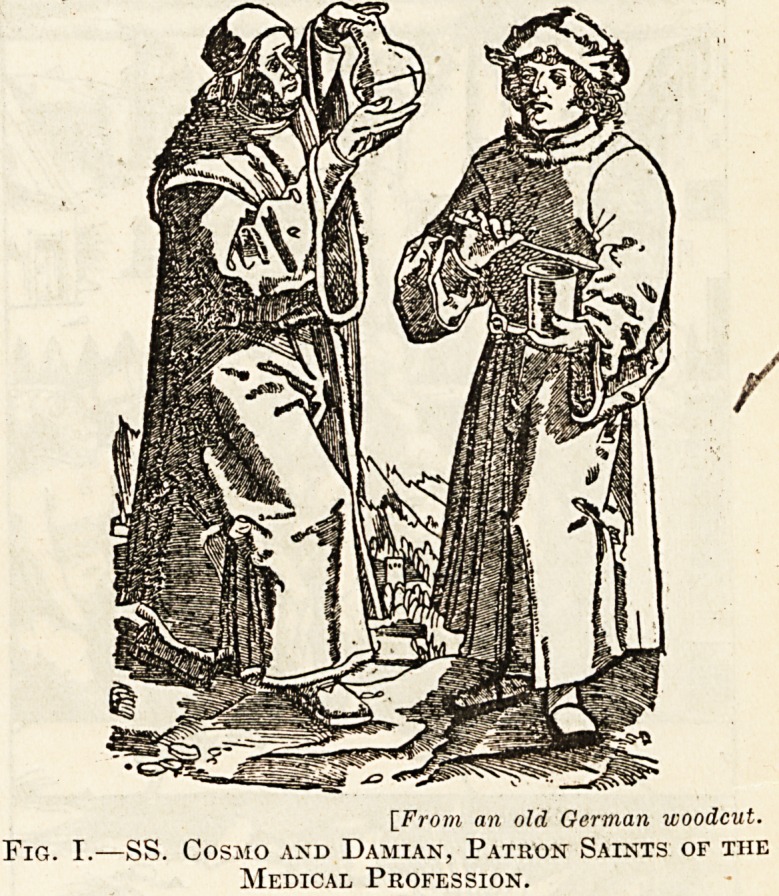


**Fig. II. f2:**
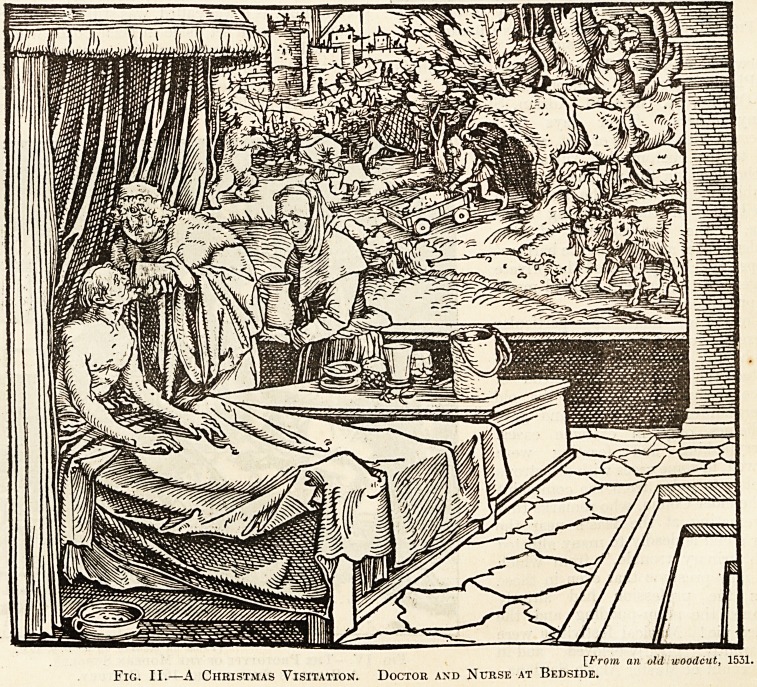


**Fig. III. f3:**
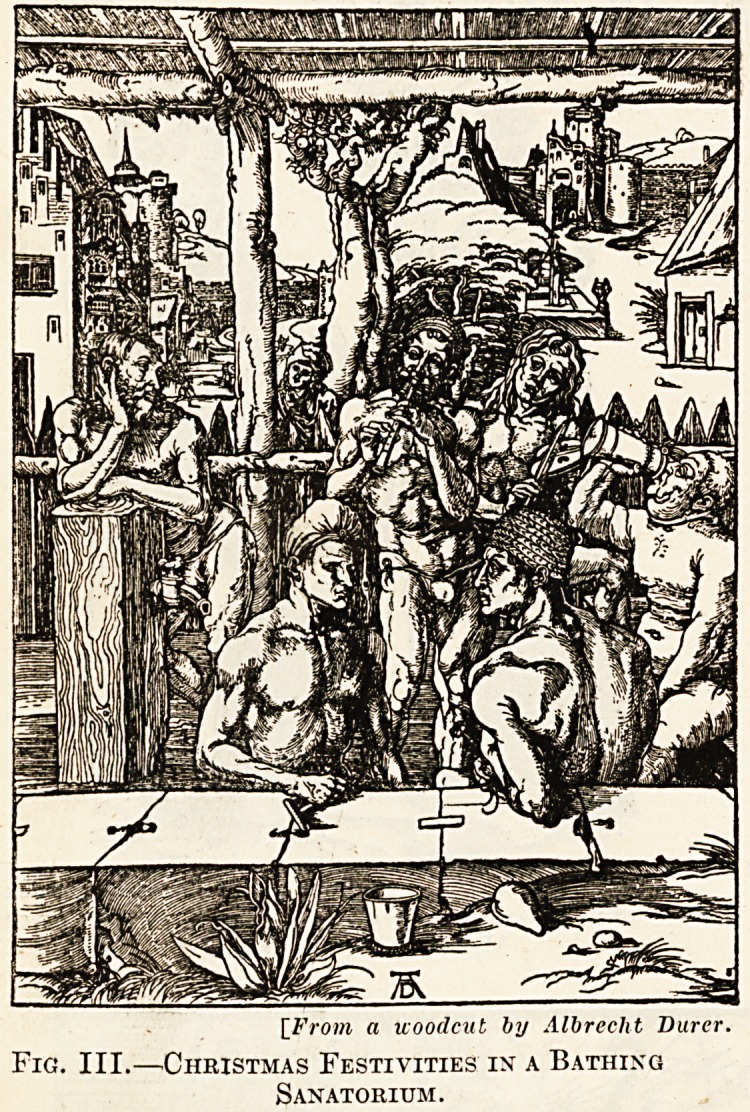


**Fig. IV. f4:**